# Ultrasound-Guided Botulinum Toxin Injections for Hand Spasticity: A Technical Guide for the Dorsal Approach

**DOI:** 10.3390/toxins17050225

**Published:** 2025-05-03

**Authors:** Calogero Malfitano, Antonio Robecchi Majnardi, Arianna Pesaresi, Vincenzo Ricci

**Affiliations:** 1 Department of Biomedical Sciences for Health, University of Milan, 20133 Milan, Italy; 2 Azienda di Servizi alla Persona Istituti Milanesi Martinitt e Stelline e Pio Albergo Trivulzio, 20146 Milan, Italy; 3 Department of Medicine, Neurology and Rehabilitation, IRCCS Istituto Auxologico Italiano, 20149 Milan, Italy; a.robecchi@auxologico.it; 4 Physical Medicine and Rehabilitation Residency Program, University of Milan, 20133 Milan, Italy; arianna.pesaresi@unimi.it; 5 Physical and Rehabilitation Medicine Unit, Luigi Sacco University Hospital, ASST Fatebenefratelli-Sacco, 20157 Milan, Italy; vincenzo.ricci58@gmail.com

**Keywords:** spasticity, hand, muscles, botulinum toxin, ultrasonography, injections

## Abstract

Spasticity often occurs following neurological disorders such as traumatic brain injury, cerebral palsy, and stroke. Botulinum toxin (BTX) injections, especially when paired with rehabilitation, are among the most effective interventions for these patients. Various techniques for administering BTX injections to the upper limb muscles have been described. However, a standardized method for ultrasound-guided injections in the intrinsic muscles of the hand remains quite scant in the literature. The authors suggest a novel dorsal approach to treat the most common abnormal postural patterns in hand spasticity, thumb-in-palm, and intrinsic plus. This approach facilitates access to the muscles while minimizing patient discomfort, as it avoids the need to open forcibly the spastic hand. The adductor pollicis, flexor pollicis brevis, lumbrical, and interosseus muscles have been identified as primary anatomical targets to improve hand posture and function. Standardized sonographic scans are leveled with anatomical illustrations and probe/patient positioning images for interventional procedures. Additionally, tips and techniques for promptly identifying vascular bundles are included to enhance the safety of the procedures. This technical report aims to provide an easy and ready-to-use tool in clinical practice for injecting intrinsic hand muscles in spastic patients, utilizing a novel dorsal approach.

## 1. Introduction

Spasticity is a frequent complication following neurological disorders such as traumatic brain injury, cerebral palsy, and stroke [1]. It is characterized by an elevated resistance to movement, increased muscle tone, and tonic tension reflexes. Excessive muscle stiffness and reduced flexibility of the myotendinous chains progressively lead to a loss of function and disability [2,3].

In the field of rehabilitation for spastic dysfunctions, one of the most effective interventional approaches involves botulinum toxin (BTX) injections [4,5]. BTX injections offer high-level precision and prolonged effectiveness by targeting only the affected muscles. Its neurotoxic features induce controlled muscle relaxation, enhancing further range of motion and mobility. Moreover, the adaptability of BTX dosages ensures a tailored approach, optimizing clinical outcomes for different spastic patterns [6]. According to the current scientific literature, a US-guided palmar injection technique is considered an optimal approach for treating spasticity of the intrinsic muscles of the hand [7,8]. Indeed, in patients who can keep the palm open, all the target muscles can be easily observed by combining longitudinal and transverse scans [9]. However, postural pathological changes of the spastic upper limb, such as forearm pronation, flexed wrist, clenched fist, and hyperextended fingers, may reduce the accessibility to specific muscles of the hand, making the interventional procedures technically challenging [10]. Forcing the hand open is not recommended since the maneuver of stretching can be painful and may exacerbate the underlying spastic pattern or trigger a grasp reflex in the hand [11]. Furthermore, spastic pattern can lead to distortion of the normal muscle anatomy [12]. Considering the clinical issues outlined above, alternative acoustic windows and injection techniques may be required. Over years of clinical experience, the authors have explored a dorsal approach to optimize the injection of intrinsic hand muscles, allowing a comfortable position for both the patient and the physician. Recently, two publications have briefly addressed the dorsal approach for the lumbricals and interossei muscles [13,14], while, to the best of our knowledge, no works in the literature describe the dorsal technique for the thenar muscle, even though it is known to be currently employed by many experts in the field.

Therefore, the aim of this guide is dual: (1) to provide a standardized, reproducible dorsal US-guided technique for injecting intrinsic hand muscles in spastic patients; (2) to highlight the clinical advantages of this method over conventional palmar approaches, in terms of anatomical accessibility, safety, and patients’ tolerability.

## 2. Results

### 2.1. Ultrasound-Guided Approach for the Thumb-in-Palm Pattern

The most common abnormal postural pattern observed in patients with upper limb spasticity involves a clenched fist [15]: flexed fingers are coupled with a curled thumb, commonly referred to as a thumb-in-palm deformity. The flexion deformity of the fingers is caused by spasticity of the flexor digitorum profundus and the flexor digitorum superficialis. The thumb-in-palm pattern is characterized by flexion and adduction of the thumb against the palm and beneath the flexed digits [16]. Key muscles contributing to this deformity are the flexor pollicis longus (FPL), the flexor pollicis brevis (FPB), and the adductor pollicis (AdP). Since the thumb is responsible for 40% of hand functions [11], its location in the palm leads to severe functional limitations for activities of daily living. Furthermore, hygiene issues may lead to palm maceration and consequent skin ulceration [17].

Since the primary objective of this manuscript is to describe the technique for performing US-guided injections of intrinsic hand muscles, the injection techniques for the forearm muscles will not be described, as standardized protocols have already been published in the pertinent literature [18,19].

Commonly, thenar eminence muscles are visualized using a palmar sonographic approach [7,9,20]. However, in spastic patients with a clenched fist and thumb-in-palm pattern, positioning the US transducer over the palmar aspect of the thenar eminence can be very challenging. Moreover, the flexed wrist and the hyperpronated forearm make injecting intrinsic hand muscles technically demanding in some patients. For these reasons, the authors suggest a dorsal approach, as it ensures access to all acoustic windows and allows the hand to maintain its postural pattern, thus preventing the forced and painful opening of the spastic hand. Furthermore, the dorsal approach can be considered less painful than the palmar one since the dorsal skin area of the hand presents fewer superficial nociceptors, as demonstrated in other interventional procedures in this anatomical region [21].

**Figure 1:**
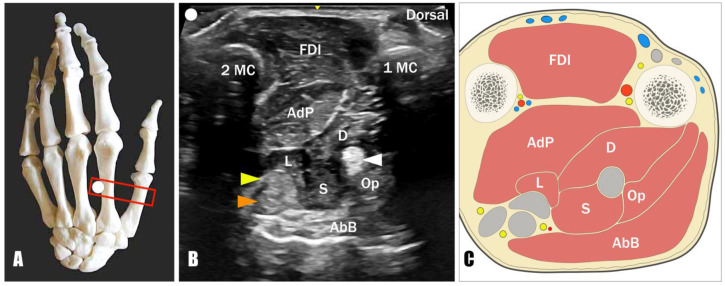
Ultrasound dorsal view and anatomical illustration of the thumb-in-palm pattern. Positioning the probe (red rectangle) in a transverse oblique plane over the dorsal aspect of the 1st intermetacarpal space (**A**), the acoustic window between the first (1MC) and second (2MC) metacarpal bone (**B**) allows the visualization of the first dorsal interosseous (FDI), the adductor pollicis (AdP), the superficial (S) and deep (D) heads of the flexor pollicis brevis, the opponens pollicis (Op), the first lumbrical muscle (L), and the abductor pollicis brevis (AbB) muscle. (**C**) Anatomical illustration of the acoustic window described in (**B**). White arrowhead: flexor pollicis longus tendon; yellow arrowhead: flexor digitorum profundus tendon of the 2nd finger; orange arrowhead: flexor digitorum superficialis tendon of the 2nd finger. Ultrasound images of an author’s hand positioned in the spastic pattern of thumb-in-palm.

The acoustic window obtained by positioning the US transducer in a transverse oblique plane over the dorsal aspect of the first intermetacarpal space enables visualization of the first dorsal interosseous (FDI), the adductor pollicis, and the flexor pollicis brevis muscle surrounding the flexor pollicis longus tendon (Figure 1).

**Figure 2:**
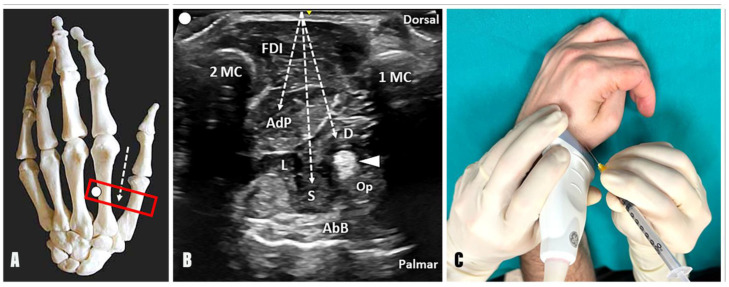
Ultrasound-guided dorsal approach for the thumb-in-palm pattern. Positioning the probe (red rectangle) in a transverse oblique plane over the dorsal aspect of the 1st intermetacarpal space (**A**), the acoustic window between the first (1MC) and second (2MC) metacarpal bone can be used to perform an out-of-plane injection (**B**,**C**) of the adductor pollicis (AdP), the superficial (S) and deep (D) heads of the flexor pollicis brevis muscle. White dotted arrow: needle’s pathway; white arrowhead: flexor pollicis longus tendon; FDI: first dorsal interosseous muscle; Op: opponens pollicis muscle; AbB: abductor pollicis brevis muscle. Ultrasound images and procedural photo of the author’s hand posed in a spastic pattern to illustrate the described technique.

Using the second metacarpal bone as a landmark, the out-of-plane technique and dorsal-to-palmar approach can be used to inject the AdP located beneath the first dorsal interosseous muscle. Likewise, by redirecting the needle towards the radial side and using the first metacarpal bone as a landmark, the superficial and deep heads of the FPB muscle can be injected with the same technique (Figure 2). For both injection techniques, a color Doppler assessment must be performed in the pre-procedural phase to locate the vascular bundles and avoid unnecessary bleeding due to microvascular injuries.

#### 2.1.1. Adductor Pollicis Muscle

- Anatomy: The AdP presents an oblique and a transverse head. The oblique component originates from the capitate bone, the second and third metacarpal bones, and the flexor carpi radialis tendon sheath; the transverse component originates from the diaphysis of the third metacarpal bone;
- Innervation: Ulnar nerve;
- Function: Adduction of the first metacarpal and flexion of the interphalangeal joint of the thumb;
- Technique: Considering the second metacarpal bone as an anatomical landmark, the needle can be advanced using an out-of-plane technique and dorsal-to-palmar approach towards the AdP by crossing the FDI (Figure 2);
- Tips and tricks: The authors suggest slightly tilting the needle in an ulnar direction after the first inoculation point into the oblique muscle belly of the AdP and advancing towards the third metacarpal bone. At this level, a second inoculation can be performed to promote the diffusion of BTX towards the transverse muscular belly of the AdP as well. Of note, a small vascular bundle can be observed between the FDI, the AdP, and the second metacarpal bone using a transverse scan over the dorsal aspect of the first intermetacarpal space. In this sense, before the aforementioned ulnar tilting of the needle to reach and inject the transverse muscular belly of the AdP, a color Doppler assessment of this region should always be performed to ensure a safe interventional procedure (Supplementary Video S1).

#### 2.1.2. Flexor Pollicis Brevis Muscle

- Anatomy: The FPB muscle presents two muscular bellies. The superficial head (sFPB) arises from the distal edge of the flexor retinaculum and the tubercle of the trapezium; the deep head (dFPB) originates from the trapezoid and the capitate bone. Both converge and insert at the base of the proximal phalanx of the thumb through the radial sesamoid bone of the first MCP joint.
- Innervation: Median nerve for the superficial head, ulnar nerve for the deep head.
- Function: Flexion of the thumb proximal phalanx on the first metacarpal, internal rotation of the first metacarpal on the trapezium.
- Technique: Considering the first metacarpal bone as an anatomical landmark, the needle can be advanced using an out-of-plane technique and dorsal-to-palmar approach toward the superficial and deep head of the FPB by crossing the FDI and the adductor pollicis muscle (Figure 2). Of note, using a dorsal approach, the deep head of the FPB is located between the AdP muscle and the FPL tendon; instead, its superficial head can be identified between the FPL tendon and the abductor pollicis brevis (AbB) muscle. In this sense, an excessive advancement of the needle in a palmar direction may be associated with an unintentional release of BTX into the AbB muscle.
- Tips and Tricks: A distal to proximal sonographic tracking of the flexor pollicis brevis may assist in differentiating its two muscular heads. Indeed, the superficial head originates from the flexor retinaculum and the tubercle of the trapezium bone; conversely, the deep head originates more medially from the trapezoid and capitate bones. When using this acoustic window in patients with a “thumb-in-palm” hand deformity, the Op muscle can be identified, in some rare cases, between the FPL tendon and the AbB muscle on the radial side (Figure 1). More commonly, the Op muscle is extremely difficult to recognize, and an unintentional spilling of BTX in the muscle may occur, without compromising the effective release of the thumb flexion [12].

### 2.2. Ultrasound-Guided Approach for the Intrinsic Plus Pattern

The intrinsic plus hand pattern is the second most common deformity after the clenched fist [22]. It is characterized by hyperflexed metacarpophalangeal (MCP) joints and hyperextended proximal interphalangeal joints, typically resembling a mild “swan-neck” deformity. This abnormal posture affects the appearance of the hand and compromises its overall dexterity and functionality [23]. The intrinsic hand muscles involved in the pattern are interossei (IO) and lumbricals. The precise origins, insertions, and even the number of these muscles can differ significantly among individuals, influencing the motor control of the fingers [24]. Anatomical studies have revealed variations in the origin of the lumbricals, with some arising from one tendon of the flexor digitorum profundus and others from two adjacent tendons, potentially impacting their force vectors on the metacarpophalangeal joints [25]. Furthermore, the interossei, responsible for abduction and adduction of the fingers, display variations in their number and size, particularly the dorsal interossei, which are typically larger and more consistent than their palmar counterparts [26]. Therefore, a thorough understanding of anatomy and adaptable ultrasound scanning may be necessary.

**Figure 3:**
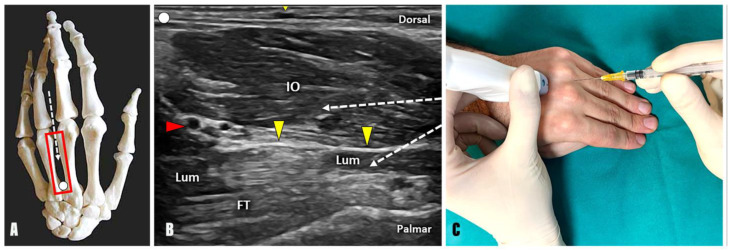
Ultrasound-guided dorsal approach for the intrinsic plus pattern. Positioning the probe (red rectangle) in a longitudinal plane over the dorsal aspect of the 3rd intermetacarpal space (**A**), the needle (white dotted arrow) can be advanced through the interdigital wing skin, using an in-plane technique and distal-to-proximal approach (**B**,**C**), injecting the lumbrical muscle (Lum) and avoiding the nearby vascular bundle (red arrowhead). IO: interosseous muscles; FT: flexor tendons; yellow arrowheads: fibrofatty band [vascular bundle: dorsal metacarpal artery and veins]. Ultrasound images and procedural photo of the author’s hand posed in a spastic pattern to illustrate the described technique.

Traditionally, by combining dorsal and palmar sonographic acoustic windows with longitudinal and transverse scanning, the dorsal and palmar interosseous muscles, as well as the lumbrical muscles, can be accurately identified using high-frequency ultrasound transducers [9]. As anticipated in two recent works [13,14], a dorsal ultrasound approach coupled with in-plane BTX injections is an accurate and safe method to treat the intrinsic plus pattern. Indeed, by positioning the US transducer over the dorsal aspect of the intermetacarpal space, the interosseous and lumbrical muscles can be recognized thanks to a thin hyperechoic fibrofatty pad interposed between them. By discerning these distinct muscle patterns, the needle can be advanced using an in-plane technique and a distal-to-proximal approach within the most superficial muscular compartment, representing the dorsal and palmar interosseous muscles, or deeper within the lumbricals (Figure 3). If necessary, a mild passive mobilization of the MCP joints can be performed to observe the differential gliding of lumbricals and interossei, optimizing their differentiation [27] (Supplementary Video S2).

#### 2.2.1. Palmar and Dorsal Interosseus Muscles

- Anatomy: The dorsal (dIO) and palmar (pIO) interossei are seven small muscles arranged in two layers between the metacarpal bones. The dIO muscles emerge from the adjacent sides of two metacarpal bones, while the pIO muscles originate from the palmar surface of the metacarpals. Both muscle groups are inserted at the extensor expansions of the fingers and the base of the proximal phalanx.
- Innervation: Ulnar nerve.
- Function: The interosseus muscles serve a dual role; dIO muscles are abductors of the fingers, whereas pIO muscles are adductors of the fingers. Moreover, IO muscles aid the lumbrical muscles in flexing the MCP joints while extending the interphalangeal.
- Technique: Positioning the probe in a longitudinal plane over the dorsal aspect of the intermetacarpal space, the needle can be advanced using an in-plane technique and a distal-to-proximal approach within the most superficial muscular compartment, which represents the dorsal and palmar interosseous muscles (Figure 3).
- Tips and tricks: Interestingly, the anatomical arrangement of the interosseous muscles within the second intermetacarpal space makes the distinction between dorsal and palmar one quite reliable, as they lie parallel to each other in the longitudinal axis. Likewise, their arrangement does not follow the same spatial pattern in the third and fourth intermetacarpal spaces, arranging themselves next to each other or twisting together. Moreover, individual anatomical variability makes the distinction of muscle boundaries even more complicated in such a small anatomical district as the intermetacarpal interval [26]. In the authors’ experience, the BTX injection of the interosseous muscle “complex” ensures accurate management of hand spasticity without the need to distinguish between the dorsal and palmar components.

#### 2.2.2. Lumbrical Muscles

- Anatomy: Lumbricals are four worm-like muscles located in the spaces between the fingers. They emerge from the flexor digitorum profundus tendons and attach to the lateral aspects of the extensor tendons from the second to the fifth fingers.
- Innervation: Median nerve for the two lumbrical muscles on the radial side and ulnar nerve for the two lumbrical muscles on the ulnar side.
- Function: Flexion of the MCP joints while simultaneously extending the interphalangeal joints.
- Technique: Positioning the probe in a longitudinal plane over the dorsal aspect of the intermetacarpal space, the needle can be advanced using an in-plane technique and distal-to-proximal approach within the lumbrical muscles (Figure 3). Interestingly, a thin hyperechoic fibrofatty band is located between the interosseous and lumbrical muscles and can be used as a sonographic landmark to optimize the accuracy of the procedure. Likewise, a dynamic scanning with passive/active mobilization of the fingers can be performed to observe a differential gliding of the interosseous and lumbrical muscles within the intermetacarpal space (Supplementary Video S2).
- Tips and tricks: Using the B-mode, a small vascular bundle can be identified within the hyperechoic fat pad between the interosseous and lumbrical muscles (Figure 3). Using color Doppler, a small vascular bundle is observed between the interosseous and lumbrical muscles within the third intermetacarpal space. Therefore, a color Doppler assessment of the intermetacarpal space should always be performed before the injection to avoid unintentional bleeding and iatrogenic injuries to the hand vasculature (Supplementary Video S3).

## 3. Discussion

The management of spasticity in the intrinsic muscles of the hand represents a complex challenge in rehabilitative medicine, demanding a nuanced understanding of neuromuscular physiology, anatomy, and precise therapeutic intervention. Traditional anatomic landmarks are not reliable in clinical practice, as they do not consider the hand’s postural abnormalities in patients with spasticity [12,28], nor the interindividual anatomical variability of intrinsic hand muscles [26,29]. Ultrasound guidance provides a non-invasive method to visualize the intrinsic muscles of the hand, such as the interossei, lumbricals, and thenar muscles, ensuring precise needle placement [30]. The enhanced accuracy provided by ultrasound guidance can lead to improved clinical outcomes, including more targeted muscle relaxation, reduced pain, and improved hand function [31].

While traditional palmar approaches offer good visualization in relaxed hands, they become technically demanding and potentially painful in spastic conditions. This gap can be overcome through the dorsal approach. Although it may be seen as more challenging for a novice sonographer, in the authors’ clinical experience, this method enables accurate injection of the target muscles without forcibly opening the hand, ensuring easy and safe anatomical access and patient tolerability. Two primary needle guidance techniques are employed: in-plane and out-of-plane. The in-plane approach involves inserting the needle parallel to the long axis of the transducer, allowing visualization of the entire needle shaft and tip throughout its course. This ensures the medication is delivered directly to the intended muscle fibers and avoids injecting it into subcutaneous fat or adjacent structures like nerves and vessels [32]. In this sense, the in-plane technique with a distal-to-proximal approach through the interdigital skin can be regarded as the safest/best technique to inject the interosseous and lumbrical muscles, as previously suggested by other authors [13,14]. However, maintaining visualization of the entire needle requires significant skill and coordination.

To inject the thenar musculature, the authors advocate using an out-of-plane approach. This recommendation is based on the limited physical space available to accommodate needle insertion beneath the transducer using an in-plane technique, as the probe must be positioned between two closely spaced bony landmarks, the first and second metacarpals. The out-of-plane technique involves inserting the needle perpendicular to the transducer face; only the needle tip (or its tissue displacement) is visualized as a hyperechoic dot. While potentially simpler to learn and perform, especially for beginners or when injecting multiple sites quickly, it provides less certainty about the exact location of the needle shaft. The principal risk lies in inadvertently going through the target muscle and infiltrating adjacent structures. Such misplacement may significantly compromise the clinical outcome of the procedure by reducing the effectiveness of the treatment [33]. To mitigate this risk, a practical and effective maneuver involves retaining a small hypoechoic droplet of BTX at the needle tip. This enhances the echogenicity of the needle path, thereby improving needle visualization during the real-time US-guided procedure. Another significant concern is the possibility of inadvertently injuring underlying vascular structures, potentially leading to hematoma formation [34]. However, it represents a manageable risk when strict adherence to safety protocols is maintained, especially through the color Doppler assessment before the interventional procedure. Indeed, the color Doppler box facilitates the identification of vascular elements, thereby reducing the risk of unintentional local bleeding. The linear transducer’s low pulse repetition frequency (0.6–0.8 KHz) should be set to visualize low-velocity blood flows and small-sized vessels (hand microvasculature).

Despite the clear advantages provided by the ultrasound guide, the final substantial benefits are contingent upon the skill and proficiency of the practitioner. Mastering USG-guided injection techniques, especially for the anatomical complexity of the region (e.g., small muscle size, depth, proximity of vital structures, potential for variability), and distortion due to spasticity, involves a distinct and acknowledged learning curve. This curve encompasses acquiring specific cognitive knowledge (sonoanatomy, ultrasound physics) and complex psychomotor skills (hand-eye coordination, probe manipulation, needle guidance). Thus, dedicated education and training are essential for the responsible and effective use of this guidance technology.

## 4. Materials and Methods

This paper is a technical report describing a novel injection technique resulting from the shared clinical experience of senior Physical and Rehabilitation Medicine physicians, each possessing advanced expertise in musculoskeletal ultrasound and clinical management of hand spasticity. We have been using this approach for at least five years, achieving good clinical outcomes and receiving positive patients’ feedback. Therefore, we plan to conduct a clinical study to outline the effects of the BTX injections using the aforementioned dorsal approach. This study proposes a step-by-step procedural guide to standardize the technique and provide tips and tricks for optimizing procedures and avoiding common technical pitfalls. Ultrasound images, anatomical correlates, and technical descriptions for the injections are educational. The figures present ultrasound images and procedural photos of one author’s hand to illustrate the described technique. Ethical committee approval was not required as no patient data were involved in developing this technical guide.

The patient should be positioned comfortably, resting on a chair or lying supine on the bed, allowing the upper limb and hand to maintain their posture pattern. Given the small size and superficial location of the target muscles, using a high-frequency ultrasound transducer, coupled with a high-end imaging system, is recommended to optimize visualization. In particular, the authors strongly advocate for using an ultrasound transducer operating within a 10–20 MHz frequency range. Nonetheless, the technique can be successfully adapted using standard 10–15 MHz linear transducers, provided that appropriate image optimization settings are applied. Employing a sterile technique, including sterile gel and transducer covers, should be considered a standard of care in ultrasound-guided procedures.

A 25-gauge needle is commonly preferred, offering an optimal balance between maneuverability and sonographic visibility throughout the procedure. The necessity of aspiration is debated, as some argue that the risk of injecting into a blood vessel is low due to the typically small-gauge needles used and that a color Doppler assessment is performed chiefly before the injection, as previously described. Treatment is highly personalized, considering the unique spasticity pattern, muscle size/volume, prior response, and desired functional outcomes. Dosing varies considerably depending on the specific BTX formulation (e.g., onabotulinumtoxinA, incobotulinumtoxinA, abobotulinumtoxinA): in our experience, each lumbricals/interossei typically receive 5–15 U inco/onaBTX-A (or 15–25 U aboBTX-A), whereas thumb muscles (adductor pollicis, flexor pollicis brevis) might receive 10–25 U inco/onaBTX-A (or 25–50 U aboBTX-A). Higher concentrations in smaller volumes are often preferred to minimize unwanted spread.

A comprehensive understanding of the anatomy, patient selection, injection techniques, and potential complications is essential for optimizing patient outcomes and minimizing adverse events. Furthermore, post-injection management, encompassing physiotherapy and occupational therapy, is crucial [35]. They target the underlying weakness, impaired motor control, and learned non-use that BTX does not directly address [36]. Indeed, integrating BTX with a structured, goal-directed rehabilitation program within a multidisciplinary team approach is essential to convert the temporary reduction in impairment into meaningful, potentially lasting functional gains and improved quality of life [37].

## Data Availability

No new data were created.

## Supplementary Materials

The following supporting information can be downloaded at: https://www.mdpi.com/article/10.3390/toxins17050225/s1, Video S1: color Doppler showing a small vascular bundle between the first dorsal interosseous muscle, adductor pollicis muscle, and second metacarpal bone; Video S2: Dynamic ultrasound assessment of the lumbrical muscles using a longitudinal scan and dorsal approach over the 3rd intermetacarpal space; Video S3: color Doppler showing a small vascular bundle between the interosseous and lumbrical muscles in the 3rd intermetacarpal space.

## Abbreviations

The following abbreviations are used in this manuscript:

BTX	Botulinum toxin
US	Ultrasound
FLP	Flexor longus pollicis
FBP	Flexor brevis pollicis
AdP	Adductor pollicis
MCP	Metacarpophalangeal
IO	Interosseus muscles
FDI	First dorsal interosseous
